# When Timing Matters: Effects of Maternal Separation and Post-Weaning High-Fat Diet on Liver Morphology in a Rodent Model

**DOI:** 10.3390/nu17101619

**Published:** 2025-05-09

**Authors:** Mariano del Sol, Javiera Navarrete, Laura García-Orozco, Jhonatan Duque-Colorado, Zaida Sócola-Barsallo, Cristian Sandoval, Bélgica Vásquez

**Affiliations:** 1 Center of Excellence in Morphological and Surgical Studies, Universidad de La Frontera, Temuco 4811230, Chile; l.garcia05@ufromail.cl (L.G.-O.); j.duque01@ufromail.cl (J.D.-C.); 2 Doctoral Program in Morphological Sciences, Faculty of Medicine, Universidad de La Frontera, Temuco 4780000, Chile; j.navarrete10@ufromail.cl; 3 Department of Health Sciences, Universidad Técnica Particular de Loja, San Cayetano Alto, Calle París, Loja 110107, Ecuador; zesocola@utpl.edu.ec; 4 Escuela de Tecnología Médica, Facultad de Salud, Universidad Santo Tomás, Los Carreras 753, Osorno 5310431, Chile; cristian.sandoval@ufrontera.cl; 5 Departamento de Medicina Interna, Facultad de Medicina, Universidad de La Frontera, Temuco 4811230, Chile; 6 Department of Basic Sciences, Faculty of Medicine, Universidad de La Frontera, Avenida Francisco Salazar 01145, Temuco 4811230, Chile

**Keywords:** maternal separation, high-fat diet, liver morphology, early-life stress

## Abstract

**Background**: Early-life stress and dietary habits are key determinants of metabolic health. This study investigates the combined effects of maternal separation (MS) and a post-weaning high-fat diet (HFD) on liver morphology in male C57BL/6 mice. **Methods**: Male mice were subjected to MS during early postnatal life or kept unmanipulated (UM). After weaning, animals were assigned to either a control diet (CD) or an HFD, forming four groups: UM-CD, UM-HFD, MS-CD, and MS-HFD. Liver histology, collagen deposition, and both morphometric and stereological parameters were assessed following 16 weeks of dietary intervention. **Results**: MS and HFD independently altered liver structure, while the combination of both factors intensified these changes. The MS-HFD group exhibited pronounced steatosis, mixed inflammatory infiltrates, and hepatocellular ballooning, with a significantly higher NAFLD Activity Score (NAS). No significant differences were observed in liver fibrosis. Morphometric analysis revealed increased body mass in HFD-fed groups and elevated liver mass in MS-HFD. Liver volume was higher in MS-HFD, though not significantly. Liver stereology revealed reduced numerical density of hepatocytes (Nv_hep_) and increased surface density (Sv_hep_) in MS groups, with the most pronounced effects in MS-HFD. **Conclusions**: Maternal separation amplifies the hepatic alterations induced by HFD, promoting early inflammatory and steatotic changes. These findings highlight the significance of early-life stress as a factor increasing susceptibility to diet-induced liver damage.

## 1. Introduction

The concept of metabolic programming proposes that exposure to environmental factors during critical periods of development can produce permanent effects on the organism’s physiology and metabolism, predisposing it to metabolic diseases in adulthood [1,2]. Among these early environmental factors, neonatal psychosocial stress, such as maternal separation, has become an essential modulator of long-term metabolic health [3].

Maternal separation (MS) is a widely used experimental model to study the effects of early-life stress in rodents. This procedure involves separating pups from their mothers for specific periods during early postnatal life, leading to alterations in the programming of the hypothalamic–pituitary–adrenal (HPA) axis that persist into adulthood [4,5]. Recent research has shown that these alterations are not limited to the central nervous system but also affect various peripheral systems, including energy metabolism and the function of metabolically active organs, such as the liver [6,7,8,9].

Growing evidence suggests early-life stress can permanently alter liver development and its functional capacity [10]. Recent studies have identified that MS induces epigenetic changes in hepatic genes related to carbohydrate and lipid metabolism, contributing to increased susceptibility to developing hepatic steatosis, non-alcoholic fatty liver disease (NAFLD), and insulin resistance in adulthood [11,12,13].

Additionally, the Western diet, characterized by high saturated fat content, has been considered one of the leading environmental factors associated with developing metabolic diseases [14]. In this sense, various studies carried out in rodent models have highlighted that exposure to high-calorie diets for specific periods of time (2–22 weeks) produces the highest serum levels of cholesterol, triglycerides, LDL cholesterol, resistance to insulin and liver enzymes (ALT and AST), severe macrovesicular hepatic steatosis and periportal inflammation [15], gradual development of NAFLD (characterized by sustained increases in ALT, HOMA-IR, serum cholesterol, and TNF-α), grade 2–3 hepatic steatosis, lobular inflammation, and the appearance of perisinusoidal fibrosis [16]. In the last study, the researchers also reported a significant increase in plasma lipopolysaccharides (LPS), suggesting an alteration in intestinal permeability and highlighting the role of the gut–liver axis in disease progression. This finding, alongside results from other studies, suggests that high-fat diets (HFDs) and foods with high energy density can induce changes in the intestinal microbiota, promoting a state of dysbiosis associated with metabolic syndrome [17]. This dysbiosis, in turn, contributes to hepatic morphological alterations linked to inflammation, steatosis, NAFLD, and other related pathologies [18].

A particularly relevant and less explored aspect is the interaction between MS and subsequent exposure to HFD, as studies have demonstrated that the combination of these two factors leads to more severe metabolic disorders than exposure to either one alone [19]. These alterations include increased mass and adiposity, glucose intolerance, fasting hyperinsulinemia, and an elevated HOMA-IR index, as well as hepatic steatosis, increased hepatic triglyceride content, and changes in the expression and phosphorylation of the glucocorticoid receptor. The latter findings suggest a dysfunction in HPA axis signaling with hepatic repercussions.

These findings reinforce the hypothesis that psychosocial stress during sensitive periods of development increases liver vulnerability to subsequent obesogenic environments. In this context, it is relevant to investigate further the effects that this combination of factors may exert on the liver’s structure during key developmental stages. Therefore, the objective of the present study was to evaluate the impact of early maternal separation on morpho-quantitative liver characteristics in mice exposed to a high-fat diet after weaning.

## 2. Materials and Methods

### 2.1. Animals

Experimental procedures were performed based on the Guide for the Care and Use of Laboratory Animals [20], and the experimental protocol was approved by the Scientific Ethics Committee of the Universidad de La Frontera, Chile (File Number 009_23) on 18 January 2023. Throughout the entire procedure, continuous monitoring measures were implemented to prevent signs of distress, dehydration, or stress, in accordance with established standards for animal welfare supervision. Female C57BL/6 mice were obtained from the Animal Facility of the Center of Excellence in Morphological and Surgical Studies (CEMyQ) at the Universidad de La Frontera, Chile. The animals were kept under controlled temperature and humidity conditions, with a 12:12 h light–dark cycle, and they had free access to food and water. Sexually mature virgin females were housed with males overnight, and observation of vaginal plugs confirmed mating. Pregnant female mice were subsequently housed individually.

During gestation and lactation, they were fed a control rodent diet (CD), AIN-93G, recommended by the American Institute of Nutrition to support gestation, lactation, and growth [21], and they had access to water ad libitum (Table 1). The diets were prepared explicitly for this study by PRAGSOLUÇÕES Biociências (www.pragsolucoes.com.br, accessed on 1 April 2025), and stored at −20 °C throughout the study for optimal preservation.

Three days before delivery, the presence of pups was checked twice daily (at the beginning and end of the light period). The day of birth was considered postnatal day 0. Sex determination of the pups was performed on postnatal day 2 by visually identifying a dark pigmented spot in the anogenital region, a characteristic exclusive to newborn males with dark pigmentation. This method allows for precise and early differentiation from female pups, which lack this pigmentation [22]. Litters were randomly adjusted to 7 pups each to ensure adequate and standardized nutrition during lactation.

### 2.2. Maternal Separation

The maternal separation protocol was conducted following the recommendations of George et al. [23], who propose that prolonged periods of maternal separation should be combined with another stressor, such as early weaning, in mice. This is because mouse dams tend to compensate intensely for separation through increased maternal behaviors, particularly enhanced grooming. Thus, early weaning strengthens the effectiveness of the model, ensuring the induction of early-life stress-related behaviors, such as anxiety and hyperactivity.

**Figure 1:**
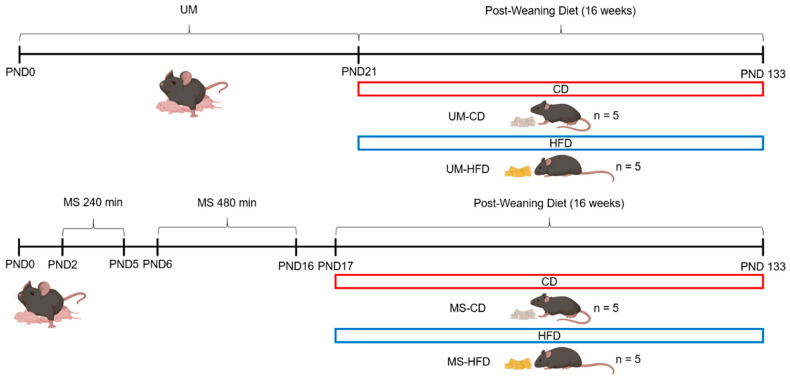
Schematic representation of the experimental design. PND: postnatal day, UM: unmanipulated, MS: maternal separation, CD: control diet, HFD: high-fat diet, UM-CD: unmanipulated group with control diet, UM-HFD: unmanipulated group with a high-fat diet, MS-CD: maternal separation group with control diet, MS-HFD: maternal separation group with a high-fat diet.

Based on this, the study groups were organized as follows: a non-manipulated (UM) group with standard weaning at 21 days and an experimental group subjected to maternal separation (MS) for 240 min per day from postnatal day 2 (the first day of separation) to day 5 and 480 min of separation from postnatal day 6 to 16, combined with early weaning on day 17 (Figure 1).

During separation periods, the pups were left in their home cage, while the dams were relocated to new cages in an adjacent room, where the same conditions of temperature, humidity, noise, cleanliness, and access to food and water ad libitum were maintained. The pups remained with their littermates throughout the separation procedure, and the cages were placed on a heating pad to maintain a constant temperature (32–34 °C) and support thermoregulation. After the separation period, the dams were returned to the home cage as appropriate. This procedure was conducted between 9:00 a.m and 1:00 p.m. or 5:00 p.m., depending on the corresponding postnatal day. Daily inspections were conducted throughout the procedure to detect signs of dehydration or distress, following the “Animal Supervision” protocol described by Morton and Griffiths [24]. Routine cage cleaning was performed by a single operator in both groups, ensuring maximum care to minimize stress during the procedure.

### 2.3. Post-Weaning Diet

At the time of weaning (postnatal day 21 for the UM group and postnatal day 17 for the MS group), one male pup per litter was randomly selected to form the experimental subgroups according to the assigned diet: control diet (CD) or high-fat diet (HFD) [25] (Table 1). Four subgroups were thus formed, UM-CD, UM-HFD, MS-CD, and MS-HFD (n = 5 mice per subgroup), with a total of 20 mice included in the study (Figure 1). Body mass was measured weekly to monitor the effect of diet consumption. Each subgroup included only male mice as the estrous cycle in females can influence behavioral variables, such as anxiety and food intake [26].

The selection of five animals per group follows widely accepted statistical and methodological criteria in morphological studies. This decision adheres to the 3R bioethical principles proposed by Russell and Burch [27] for animal experimentation (replacement, reduction, and refinement). From a statistical perspective, Cruz-Orive and Weibel [28] note that selecting five animals per group provides a solid starting point. If a consistent trend emerges, the probability that this result is due to chance is P = (1/2)^5^ = 0.03125, which suggests that the finding may be considered potentially conclusive in exploratory studies.

### 2.4. Euthanasia

After completing the observations (16 weeks post-weaning), the mice were fasted for six hours. The mass of each animal was recorded prior to euthanasia, which was performed using an overdose of 240/30 mg/kg ketamine/xylazine [29]. The liver was then dissected to determine its mass using an analytical balance (A&D Orion^®^ HR 120, A&D Technology, Saitama, Japan) and its volume using the Scherle method [30]. Several liver fragments were immersed in fixative (1.27 mol/L formaldehyde in 0.1 M phosphate buffer, pH 7.2) for 48 h at room temperature.

### 2.5. Histological Processing and Staining

Once fixed, the samples were dehydrated and embedded in Paraplast Plus (Sigma-Aldrich Co., St. Louis, MO, USA). Then, 5 μm thick sections were obtained from the resulting blocks using a microtome (Leica^®^ RM 2255, Leica Biosystems, Deer Park, IL, USA). Five sections were made from each block for histopathological and stereological analysis. Sections were stained with hematoxylin and eosin (H&E) to examine tissue structure and with Sirius Red to identify and measure type I and III collagen fibers. Histological images were captured using a Leica^®^ DM750 microscope (Leica Microsystems, Heerbrugg, Switzerland) equipped with a Leica^®^ ICC50 HD digital camera (Leica Microsystems, Heerbrugg, Switzerland) and projected onto a ViewSonic^®^ LCD screen (ViewSonic Corporation, Brea, CA, USA).

### 2.6. Histopathological Evaluation

To evaluate the histological activity of non-alcoholic fatty liver disease (NAFLD), the NAFLD Activity Score (NAS) was used—a semi-quantitative system developed by the Pathology Committee of the NASH Clinical Research Network (NASH CRN) and validated by Kleiner et al. [31]. This index standardizes the assessment of disease severity based on three primary parameters: steatosis grade (0–3), lobular inflammation (0–3), and hepatocellular ballooning (0–2), with a total score ranging from 0 to 8. Histological analysis was conducted on H&E-stained sections by two independent evaluators (ZSB and BV) blinded to the identity of the experimental groups. Discrepancies in scoring were resolved through consensus. According to Kleiner et al. [31], a NAS score ≥ 5 is considered highly suggestive of non-alcoholic steatohepatitis (NASH), while a score < 3 indicates a low probability of NASH.

### 2.7. Collagen Fiber Quantification

To quantify type I and III collagen fibers in the liver, histological sections were stained with 0.1% *w*/*v* Sirius Red F3BA (Sigma-Aldrich Co., St. Louis, MO, USA) for 1 h in a saturated aqueous solution of picric acid (Merck, Darmstadt, Germany). They were then rinsed with 0.01 N hydrochloric acid (Merck, Darmstadt, Germany) for 2 min, washed with distilled water, counterstained with Harris hematoxylin (Merck, Darmstadt, Germany) for 2 min, and rinsed with running water. Finally, the sections were dehydrated in ascending alcohols, cleared in xylene (Merck, Darmstadt, Germany), and mounted with Entellan^®^ (Merck, Darmstadt, Germany). Histological images were obtained with a Leica^®^ DM750 microscope and a Leica^®^ ICC50 HD camera and projected onto a ViewSonic^®^ LCD screen (ViewSonic Corporation, Brea, CA, USA). The total area (μm^2^) of type I and III collagen fibers was measured using Image Pro Premier 9.1 software (Media Cybernetics, Warrendale, PA, USA).

### 2.8. Liver Stereology

For liver stereological analysis, five random microscopic fields were examined for each histological section, resulting in a total of 240 investigated fields (120 per group) [32]. The slides were examined using a Leica^®^ DM2000 LED stereological microscope (Leica Microsystems, Heerbrugg, Switzerland) and captured with a Leica^®^ MC170 HD digital camera (Leica Microsystems, Heerbrugg, Switzerland). The 36-point testing technique developed by STEPanizer^®^ software for quantitative histological analysis (version 2.28, https://www.stepanizer.com/, accessed on 10 March 2025) was employed to ascertain the following parameters: volume density (Vv_hep_), surface density (Sv_hep_), and quantity (Nv_hep_) of hepatocyte nuclei. Volume density was calculated using the formula Vv = P_P_/P_T_ (×100%), where P_P_ represents the number of points impacting the structure of interest and P_T_ denotes the total number of points in the test system (36 points). Surface density was determined using the formula Sv = 2 × I/L_T_, where I represents the number of intersections with the structure of interest and L_T_ is the total test line length inside of the system. The numerical density was determined using the equation Nv = Q^−^/(A_T_ × t), where Q^−^ represents the count of observed profiles of the structure of interest within a certain region, including banned lines and planes, A_T_ denotes the overall test area, and t signifies the dissector thickness. The slides were examined using a Leica^®^ DM2000 LED stereomicroscope and captured with a Leica^®^ MC170 HD digital camera.

### 2.9. Statistical Analysis

Levene’s test was used to evaluate the homogeneity of variances, whereas the Shapiro–Wilk test was used to analyze data normality. These tests were utilized to facilitate the precise analysis of the quantitative data. Group disparities were evaluated using one-way ANOVA or, when appropriate, the Kruskal–Wallis test. Post hoc analyses, including Dunnett’s T3 and Tukey’s HSD, were performed as necessary. A *p*-value under 0.05 was considered statistically significant. All analyses were conducted using IBM SPSS Statistics, version 21 (IBM Corp., Armonk, NY, USA).

## 3. Results

### 3.1. Maternal Separation and a High-Fat Diet Induce Liver Alterations, Evidenced by Steatosis, Inflammation, and Hepatocellular Ballooning, with Greater Severity Observed in the Group Exposed to Both Conditions

**Figure 2:**
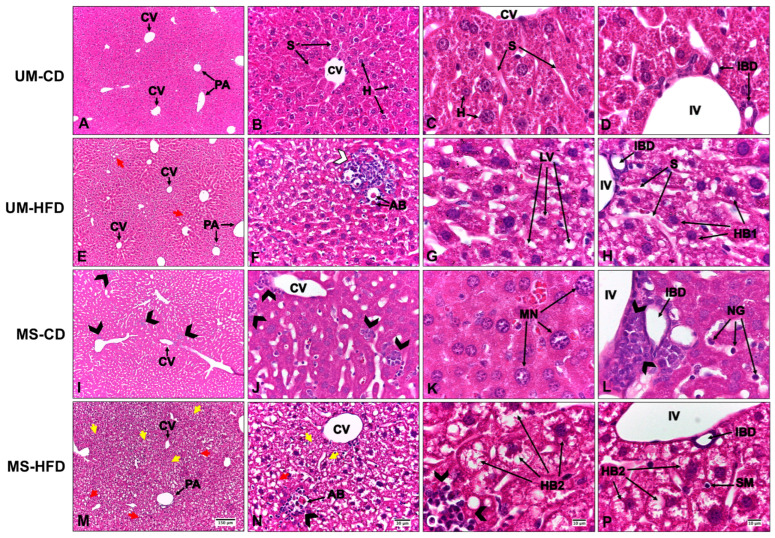
Histology of livers of C57BL/6 mice subjected to maternal separation and a post-weaning high-fat diet. UM-CD: unmanipulated group with control diet (**A**–**D**), UM-HFD: unmanipulated group with a high-fat diet (**E**–**H**), MS-CD: maternal separation group with control diet (**I**–**L**), MS-HFD: maternal separation group with a high-fat diet (**M**–**P**). CV: central vein, PA: portal area, IV: interlobular vein, IBD: interlobular bile duct, H: hepatocyte, S: sinusoid, red arrow: macrovesicular hepatic steatosis, yellow arrow: microvesicular hepatic steatosis, white arrowhead: lymphocyte-predominant leukocytes, black arrowhead: leukocytes with a predominance of neutrophils, SM: stellate macrophage, MN: macronucleus, NG: neutrophilic granule, AB: acidophilic body, LV: lipid vacuoles, HB1: grade 1 hepatocellular ballooning, HB2: grade 2 hepatocellular ballooning. H&E staining.

A well-preserved hepatic parenchyma was observed in the UM-CD group, characterized by the radial arrangement of hepatocytes around the central vein. These cells exhibited eosinophilic cytoplasm and rounded nuclei with loose chromatin, with some showing prominent nucleoli. Hepatic sinusoids were lined with fenestrated endothelial cells and occasionally contained scattered stellate macrophages throughout the parenchyma. The organization of hepatic cords was homogeneous, with no evidence of structural alterations, fibrosis, or inflammatory infiltration. The portal spaces showed a typical arrangement, with a well-defined portal triad composed of a vein, an artery, and an interlobular bile duct with a simple cuboidal epithelium. According to the NAS scoring system, all three analyzed parameters (steatosis grade, lobular inflammation, and hepatocellular ballooning) scored 0, indicating the absence of significant histological alterations (Figure 2A–D, where normal hepatic architecture without signs of inflammation or lipid accumulation is observed).

In the UM-HFD group, the hepatic parenchyma retained its histological architecture, with evidence of macrovesicular hepatic steatosis. Lipid macrovacuoles were observed partially displacing hepatocytes. In zone 3, aggregates of mononuclear leukocytes, predominantly lymphocytes, were identified, along with acidophilic bodies. NAS scoring revealed grade 1 steatosis, paracinar and non-microvesicular distribution, grade 1 inflammation, and grade 1 hepatocellular ballooning. No alterations were observed in the other evaluated parameters. In Figure 2E–H, red arrows indicate lipid vacuoles, and inflammatory infiltrates are visible in centrilobular areas.

The MS-CD group demonstrated an inflammatory infiltrate composed of mixed leukocytes—predominantly neutrophils with occasional eosinophils—in the centrilobular region. This finding suggests an acute-phase inflammatory response secondary to increased cellular injury. In addition, congestion was evident in the interlobular veins of the hepatic triads. NAS scoring indicated grade 0 steatosis and grade 2 inflammation, with no alterations in the other parameters. These features are evident in Figure 2I–L, where black arrowheads highlight neutrophilic infiltration.

In the MS-HFD group, macrovesicular and microvesicular hepatic steatosis were observed, accompanied by an inflammatory infiltrate of mixed leukocytes located interstitially and in the centrilobular region, along with ballooned hepatocytes. NAS scoring indicated grade 1 steatosis with a predominantly microvesicular pattern, grade 2 inflammation, and grade 2 hepatocellular ballooning. Occasional acidophilic bodies were identified, with no alterations in the other evaluated parameters. In Figure 2M–P, small vacuoles characteristic of microvesicular steatosis are observed (yellow arrows), as well as grade 2 ballooned hepatocytes (HB2).

No hepatic fibrosis was observed in any of the groups, and the quantification of type I and III collagen fibers did not show significant differences (Table 2).

### 3.2. Maternal Separation and Post-Weaning High-Fat Diet Affect Liver Morphometry and Stereology, Revealing Changes in the Organization and Cellular Composition of Hepatic Tissue

Statistically significant differences were observed in body mass (*p* = 0.013) and liver mass (*p* = 0.035), while liver volume did not show significant differences between groups (*p* = 0.081). Post hoc comparisons indicated that the UM-HFD group exhibited a significant increase in body mass compared to the UM-CD group. In contrast, the MS-HFD group differed from the UM-CD and MS-CD groups. Regarding liver mass, the MS-HFD group had significantly higher values than the UM-HFD group (Table 3).

The evaluated stereological parameters revealed significant differences in liver morphology among the experimental groups. The volume density of hepatocytes (Vv_hep_) showed no significant differences between groups (*p* = 0.184), with values remaining relatively constant regardless of diet or maternal separation.

In contrast, the surface density of hepatocytes (Sv_hep_) showed significant differences between groups (*p* < 0.001). An increase in Sv_hep_ was observed in the groups subjected to maternal separation (MS-CD and MS-HFD) compared to the unmanipulated groups (UM-CD and UM-HFD), suggesting an impact of maternal separation on the liver’s structure.

On the other hand, the numerical density of hepatocytes (Nv_hep_) showed a progressive reduction associated with both the high-fat diet and maternal separation, with significant differences among the groups (*p* < 0.001). The most significant reduction in Nv_hep_ was observed in the MS-HFD group (7836.26 ± 4732.54 mm^−3^), with significant differences compared to all other groups (*p* < 0.05) (Table 4).

## 4. Discussion

The findings of the present study support the hypothesis that early exposure to psychosocial stress, represented by MS, combined with an HFD after weaning, induces structural and functional alterations in the liver tissue of male C57BL/6 mice, even in the absence of detectable fibrosis. These alterations encompass histological organization and morphometric and stereological parameters that reflect a progressive dysfunction of the hepatic parenchyma.

From a histological perspective, the high-fat-diet-induced mild macrovesicular steatosis, combined with MS, intensified the alterations, including mixed inflammatory infiltrate, hepatocellular ballooning, and acidophilic bodies. These results are consistent with previous studies in experimental models that demonstrated how MS exacerbates vulnerability to obesogenic stimuli, promoting a proinflammatory and steatotic hepatic phenotype [19,33]. In addition, similar findings have been reported in human studies, where early-life adversity has been associated with metabolic disorders, increased hepatic fat accumulation, and inflammation later in life [34,35]. The increased NAS score in the MS-HFD group indicates a progression toward NASH, even in the early stages of postnatal development.

The absence of significant fibrosis in our model, despite the observed chronic inflammation, could be explained by the relatively moderate duration of the experimental protocol (16 weeks post-weaning), which represents an early or intermediate phase in the progression of liver disease. In a study on rats, Maciejewska et al. [16] demonstrated that hepatic fibrosis began to manifest after 12 weeks of exposure to a high-fat, high-cholesterol diet, reaching a higher degree of severity only after 20 weeks, suggesting that prolonged exposure is required to activate profibrotic pathways fully. In line with this, Sadana et al. [36], using C57BL/6 mice (the same model as in our study), observed that a high-fat diet over four weeks induced early metabolic and inflammatory changes but without the development of steatosis or fibrosis, highlighting that these structural changes require more prolonged exposure. Altogether, these previous findings support the idea that although the 16-week duration generates a proinflammatory environment and subtle remodeling of liver tissue, such as the slight increase of type I collagen in the MS-CD and MS-HFD groups, it is insufficient to trigger established fibrosis. These results suggest incipient extracellular matrix remodeling that could progress to fibrosis with prolonged exposures or additional stimuli.

The morphometric results indicate that both the high-fat diet and MS significantly impact body and liver mass, particularly in the group exposed to both conditions (MS-HFD). The increased body mass observed in the UM-HFD and MS-HFD groups aligns with previous studies reporting the obesogenic effect of high-fat diets, especially when administered from early developmental stages [36]. This diet promotes a positive energy balance and alters lipid and insulin metabolism, facilitating fat accumulation in the body and the liver. However, the fact that the MS-C group did not demonstrate increased body mass suggests that maternal separation, without a nutritional challenge, is insufficient to induce this phenotype. This finding is consistent with what Paternain et al. [37] reported, who observed that MS does not induce an obese phenotype per se but rather predisposes individuals to an exacerbated response to hypercaloric diets in adulthood.

The increase in liver mass explicitly observed in the MS-HFD group compared to UM-HFD suggests a synergistic interaction between early stress and the hypercaloric diet. This synergy could be due to metabolic programming induced by maternal separation, which sensitizes the liver to subsequent lipotoxic stimuli, promoting adaptive changes, such as hepatocellular hypertrophy, lipid accumulation, or expansion of inflamed tissue [12]. Although liver volume did not show statistical differences, the upward trend in the MS-HFD group may reflect initial tissue expansion that is not yet morphometrically consolidated but indicative of progressing structural changes. Altogether, these findings highlight how adverse experiences during critical stages of development can modulate the body’s response to obesogenic diets, affecting liver morphology even before the appearance of advanced histological lesions.

Regarding the stereological changes, the increase in Sv_hep_ in the MS groups may reflect greater complexity or irregularity in the cell membrane consistent with adaptive responses to cellular damage or alterations in the hepatic sinusoidal architecture. Simultaneously, the progressive decrease in Nv_hep_, especially in the MS-HFD group, may indicate compensatory cellular hypertrophy or polyploidization processes—well-known mechanisms in hepatotoxicity and liver regeneration [38]. Additionally, the reduction in Nv_hep_ could be associated with a loss of the liver’s proliferative capacity, possibly mediated by disruptions in the signaling of factors like HNF4α, whose deregulation has been linked to lipid accumulation and hepatic inflammation [39].

The synergistic effects observed between early stress and a high-fat diet support the theory of metabolic programming, demonstrating how perinatal events shape the organism’s response to future metabolic challenges [1,2]. This phenomenon may involve persistent epigenetic alterations in key genes for liver metabolism, as described in previous studies with the CD36 gene [12,13], as well as in inflammatory and oxidative stress signaling pathways [40]. In recent years, special attention has been given to microRNAs, which have emerged as both biomarkers and potential therapeutic targets in liver and gastrointestinal diseases [41].

While the maternal separation model combined with early weaning effectively induced an early stress phenotype, future investigations should include molecular analyses to elucidate the underlying mechanisms. These should include assessments of oxidative stress markers, expression of genes related to fibrogenesis (TGF-β, Col1a1), and the activity of pathways like Notch and Wnt/β-catenin, which have been implicated in liver maturation and damage response [42,43,44,45].

This study provides insights into the interaction between early-life psychosocial stress and post-weaning dietary habits, demonstrating their combined effects on liver morphology. The use of C57BL/6 mice, a well-established model for metabolic research, allows for a controlled evaluation of these factors. The study’s histological, morphometric, and stereological assessments strengthen the understanding of early hepatic alterations linked to metabolic dysfunction.

The present study has several limitations. First, the absence of molecular analyses and complementary histological evaluations, such as the characterization of Kupffer cell infiltration, alterations of the biliary tract, or sinusoidal capillarization, limited a more detailed exploration of the cellular and tissue mechanisms involved in the progression of liver pathology. Additionally, the duration of the experiment may not have been sufficient to detect advanced-stage fibrosis or chronic liver dysfunction, as these processes typically require longer exposure periods to obesogenic stimuli for their full manifestation. Furthermore, the restriction of the study to male mice limits the understanding of how sex-specific factors modulate early-life stress responses, thereby reducing the generalizability of the findings. Consequently, future studies should consider longer observation periods, incorporate molecular analyses targeting inflammatory, oxidative stress, and tissue remodeling pathways, evaluate specific alterations in the hepatic architecture, and include both sexes to achieve a more comprehensive and nuanced understanding of the effects of maternal separation and its interaction with obesogenic diets.

## 5. Conclusions

In conclusion, the results obtained indicate that the interaction between neonatal psychosocial stress and a post-weaning obesogenic diet significantly alters the structure and cellular dynamics of the liver, promoting a pro-steatotic and proinflammatory tissue environment that may precede the development of fibrosis and chronic liver dysfunction in later stages. In particular, the observed stereological changes, such as the reduction in hepatocyte numerical density and the increase in their surface density in the groups subjected to maternal separation, together with the histological alterations reflected in higher NAS scores in the MS-HFD group, suggest that maternal separation increases the liver’s sensitivity to the adverse effects of a high-fat diet. These findings underscore the importance of considering early-life events as critical determinants of long-term liver health.

## Figures and Tables

**Table 1:** Diets were administered according to the experimental group. Control diet (CD) and high-fat diet (HFD). Vitamin and mineral mixes were formulated according to the AIN-93G standard of the American Institute of Nutrition for rodents. The high-fat diet provided 49% of the energy through lipids.

Ingredients	CD	HFD
Casein (>85% protein)	200.0	230.0
L-cystine (g/kg)	3.0	3.0
Cornstarch (g/kg)	529.486	299.472
Sucrosa (g/kg)	100.0	100.0
Soybean oil (g/kg)	70.0	70.0
Lard (g/kg)	-	200.0
Fiber (g/kg)	50.0	50.0
Vitamin mixture (g/kg) ^1^	10.0	10.0
Mineral mixture (g/kg)	35.0	35.0
Choline bitartrate (g/kg)	2.5	2.5
Antioxidant (g/kg)	0.014	0.028
Total (g)	1000.0	1000.0
Energy (kcal/g)	3.95	4.95
Carbohydrate (% Energy)	64.0	32.0
Protein (% Energy)	19.0	19.0
Lipid (% Energy)	17.0	49.0

^1^ Without vitamin D.

**Table 2:** Quantifying collagen content in liver tissue of male C57BL/6 mice subjected to maternal separation and a post-weaning high-fat diet using Image Pro Premier.

	Media ± SD				
Collagen	UM-CD	UM-HFD	MS-CD	MS-HFD	*p*-Value
Type I (µm^2^)	2.16 ± 0.62	2.37 ± 0.63	2.75 ± 0.66	2.62 ± 0.58	0.079
Type III (µm^2^)	1.35 ± 0.22	1.32 ± 0.18	1.18 ± 0.31	1.24 ± 0.33	0.180

Significant differences (*p* < 0.05). UM-CD: unmanipulated group with control diet, UM-HFD: unmanipulated group with a high-fat diet, MS-CD: maternal separation group with control diet, MS-HFD: maternal separation group with a high-fat diet.

**Table 3:** Body mass, liver mass, and liver volume in male C57BL/6 mice subjected to maternal separation and a post-weaning high-fat diet.

	Median ± SD				
Variables	UM-CD	UM-HFD	MS-CD	MS-HFD	*p*-Value
Body mass (g)	25.28 ± 2.09	30.76 ± 1.71 ^a^	24.39 ± 5.79 ^b^	30.10 ± 1.55 ^a,c^	0.013
Liver mass (g)	1.56 ± 0.21	1.31 ± 0.07	1.42 ± 0.27	1.81 ± 0.36 ^b^	0.035
Liver volume (mL)	1.46 ± 0.28	1.28 ± 0.10	1.36 ± 0.29	1.74 ± 0.35	0.081

^a^ Significant differences (*p* < 0.05) with the UM-CD group. ^b^ Significant differences (*p* < 0.05) with the UM-HFD group. ^c^ Significant differences (*p* < 0.05) with the MS-CD group. UM-CD: unmanipulated group with control diet, UM-HFD: unmanipulated group with a high-fat diet, MS-CD: maternal separation group with control diet, MS-HFD: maternal separation group with a high-fat diet.

**Table 4:** Stereological parameters of hepatocytes in male C57BL/6 mice subjected to maternal separation and a high-fat diet after weaning.

	Media ± SD				
Variables	UM-CD	UM-HFD	MS-CD	MS-HFD	*p*-Value
Vv_hep_ (%)	7.57 ± 1.89	7.06 ± 2.84	7.88 ± 3.97	7.93 ± 4.08	0.184
Sv_hep_ (mm^−1^)	29.19 ± 11.98	32.87 ± 14.89	41.94 ± 18.97 ^a,b^	40.15 ± 15.95 ^a,b^	<0.001
Nv_hep_ (mm^−3^)	15,217.52 ± 5495.84	12,447.95 ± 6305.92 ^a^	12,323.15 ± 5326.38 ^a^	7836.26 ± 4732.54 ^a,b,c^	<0.001

^a^ Significant differences (*p* < 0.05) with the UM-CD group. ^b^ Significant differences (*p* < 0.05) with the UM-HFD group. ^c^ Significant differences (*p* < 0.05) with the MS-CD group. Vv_hep_: hepatocyte nuclear volume density, Sv_hep_: Hepatocyte Nuclear Surface Density, Nv_hep_: Hepatocyte Nuclear Number Density, UM-CD: unmanipulated group with control diet, UM-HFD: unmanipulated group with a high-fat diet, MS-CD: maternal separation group with control diet, MS-HFD: maternal separation group with a high-fat diet.

## Data Availability

The original contributions presented in this study are included in the article. Further inquiries can be directed to the corresponding authors.

## Abbreviations

The following abbreviations are used in this manuscript:

MS	Maternal separation
UM	Unmanipulated
HFD	High-fat diet
CD	Control diet
UM-CD	Unmanipulated group with control diet
UM-HFD	Unmanipulated group with a high-fat diet
MS-CD	Maternal separation group with control diet
MS-HFD	Maternal separation group with a high-fat diet
Vv_hep_	Hepatocyte nuclear volume density
Sv_hep_	Hepatocyte Nuclear Surface Density
Nv_hep_	Hepatocyte Nuclear Number Density
